# Customized low-cost high-throughput amplifier for electro-fluidic detection of cell volume changes in point-of-care applications

**DOI:** 10.1371/journal.pone.0267207

**Published:** 2022-04-20

**Authors:** Saurabh Kaushik, Prabhakaran Selvanathan, Gautam Vivek Soni

**Affiliations:** Raman Research Institute, Bangalore, INDIA; University of Newcastle, AUSTRALIA

## Abstract

Physical parameters of the pathogenic cells, like its volume, shape, and stiffness, are important biomarkers for diseases, chemical changes within the cell, and overall cell health. The response of pathogenic bacteria and viruses to different chemical disinfectants is studied widely. Some of the routinely employed techniques to measure these changes require elaborate and expensive equipment which limits any study to a non-mobile research lab facility. Recently, we showed a micropore-based electro-fluidic technique to have great promise in measuring subtle changes in cell volumes at high throughput and resolution. This method, however, requires commercial amplifiers, which makes this technique expensive and incompatible for in-field use. In this paper, we develop a home-built amplifier to make this technique in-field compatible and apply it to measure changes in bacterial volumes upon exposure to alcohol. First, we introduce our low-cost and portable transimpedance amplifier and characterize the maximum range, absolute error percentage, and RMS noise of the amplifier in the measured current signal, along with the amplifier’s bandwidth, and compared these characteristics with the commercial amplifiers. Using our home-built amplifier, we demonstrate a high throughput detection of ~1300 cells/second and resolve cell diameter changes down to 1 μm. Finally, we demonstrate measurement of cell volume changes in E. coli bacteria when exposed to ethanol (5% v/v), which is otherwise difficult to measure via imaging techniques. Our low-cost amplifier (~100-fold lower than commercial alternatives) is battery-run, completely portable for point-of-care applications, and the electro-fluidic devices are currently being tested for in-field applications.

## Introduction

Diagnosis of a disease is the first critical step for finding a cure for a patient [1–4]. Lack of in-field diagnostic facilities or diagnosis tools has caused numerous casualties in past [5–7]. The diagnostic industry includes X-ray, flow cytometry, ultrasound, coagulation analyzers, CT-scan, MRI, cell counters, micro sedimentation centrifuges, platelet aggregometer, enzyme assay kits, and many associated consumables [8–22]. The urban population has access to these facilities via multi-specialist hospitals, door-to-door ambulance services, private clinics, and even online medical assistance, whereas in many rural areas around the globe, the nearest medical help or essential medical equipment are miles away [23–26]. Diseases like Tuberculosis, Tetanus, Cholera, Anthrax, Pneumonia, etc., are caused by bacterial infection, which has been fatal for centuries [27–36]. Certain alcohol-based disinfectants are used to kill bacterial cells and can help prevent infections [37, 38]. Disinfectants cause denaturation, and the bacterial cells lose their structural integrity by the breakdown of membrane proteins [39–42]. There are pathogens that resist certain disinfectants [43–46], and hence a quantitative study of alcohol-based physiological changes can lead to a better understanding of how these cells evolve to develop such resistance.

In this work, we present our electro-fluidic device to measure alcohol-dependent changes in bacterial cells. This device makes a high resolution and high throughput electrical measurement which directly corresponds to changes in cell volume. We start with introducing a low-cost portable amplifier (referred to as “lab-amplifier”) customized with a microfluidic platform which is easy to build, plug and play in use, portable to be used in the field, and about a 100-fold lower in cost than the existing commercial amplifiers. Accurate electrical readout of cell volume changes is recorded with the lab amplifier with high resolution as the cells translocate through the microfluidic device (micropore). The calibration of the micropore device to estimate the volume is based on resistive pulse technique [47–56]. The lab amplifier is characterized for the maximum range, absolute error, and RMS noise in the measured current signals. We have two series of lab amplifiers (L1 and L10 series), and both are characterized to have different bandwidths with different current gains. Lab amplifier with higher bandwidth (L1 series) works better for higher throughput but at the cost of slightly higher noise than the L10 series. L1 series amplifiers are more suitable for larger cells with large resistive pulse signals [48], whereas the L10 series amplifiers are more suitable to measure subtle changes in cell volumes with higher resolution. All the electrical characterization of the lab amplifier is compared with two commercial amplifiers under identical experimental conditions. Using model cells, we demonstrate resolution and high throughput detection of up to ~1300 model cells/sec using our lab amplifier. Finally, we apply our device to quantitatively measure volumetric changes in cells (*E*. *Coli* bacteria) caused due to mild ethanol exposure in the suspension buffer. These subtle changes in cell physiology were not detectable using typical fluorescence or bright field microscopy imaging but were resolved successfully using our electro-fluidic device with lab-amplifier-based detection. There have been previous reports of making customized amplifiers for resistive pulse sensing, however, those designs are not suitable for measurement in cellular changes as they are aimed at molecular detection insteads [57–63]. Given the custom design of our lab amplifiers, we have made our portable measurement system low-cost, high throughput, and demonstratively aimed towards measuring changes in cell sizes. We foresee its multiple applications in hospitals and in-field rural settings.

## Material and methods

**Fig 1:**
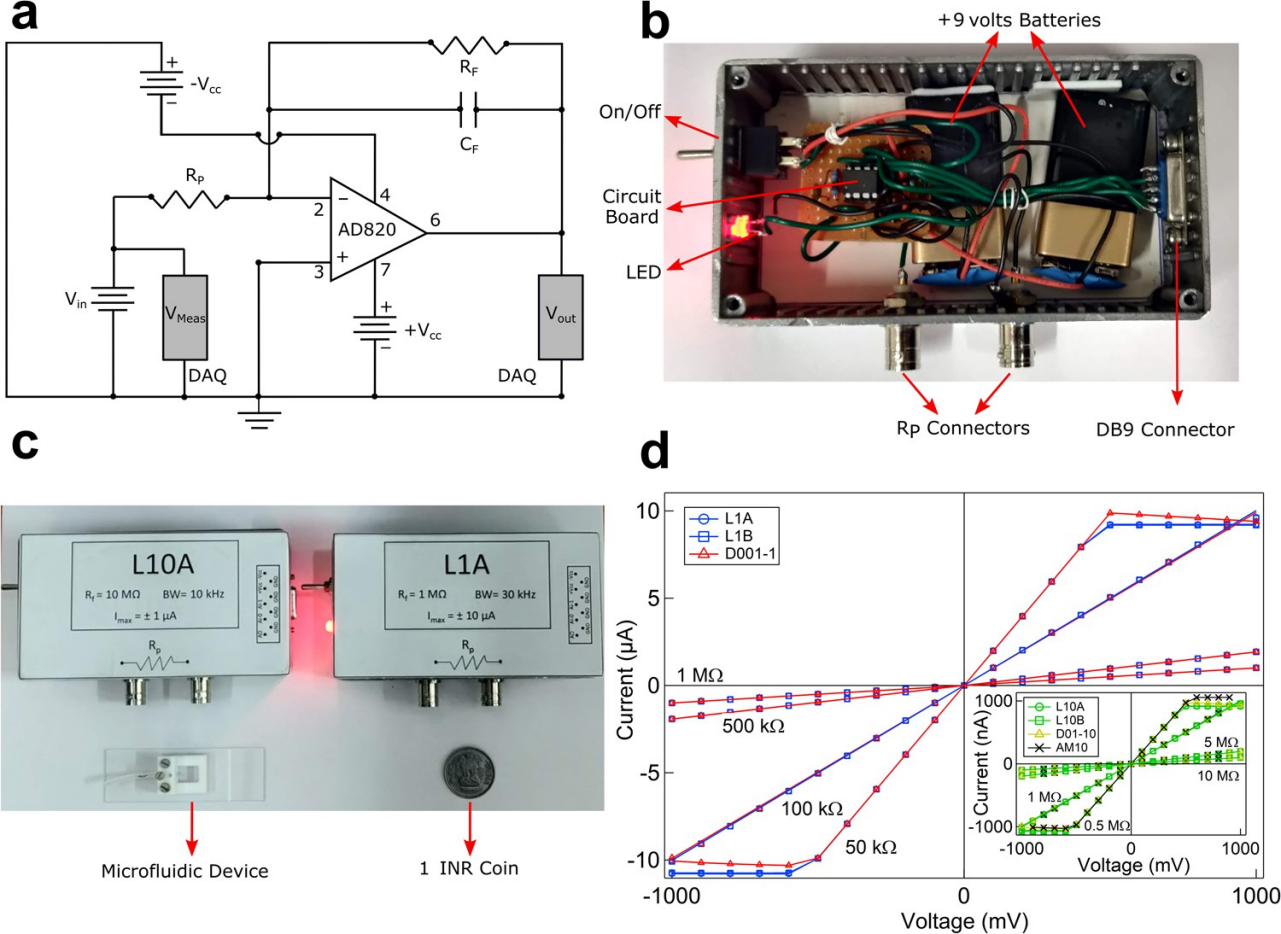
Schematic and construction of low-cost portable amplifier. **a** Schematic of the electrical circuit of the amplifier. **b** Image of the soldered circuit board inside the amplifier box with labeled circuit board, On/Off switch, LED, R_P_ connectors (to device) and DB9 connector. The amplifier is powered by two 9 volts DC batteries. **c** Shows two lab amplifiers (L1 and L10 series), enclosed in an aluminum box with labels showing individual specifications. The micropore device on a glass slide and a 1 INR coin is also shown for size reference. **d** I-V characteristics of electrical resistors measured with our amplifiers (L1 and L10 (inset), with ± 10 volts V_CC_) series where A & B suffix are 2 copies of the same amplifier design) is compared with commercial amplifiers (D and AM series). Online version in colour.

Our custom lab amplifier is designed with an inverting mode operational amplifier (Op-amp) (IC AD820) which is powered by two 9V DC batteries (+V_cc_ and -V_cc_) at IC pins 7 and 4, respectively. The feedback resistor (R_F_) and capacitor (C_F_) are connected across pins 2 and 6 of the Op-amp in parallel. We use a data acquisition card (National Instruments, NI myDAQ) to apply a constant DC input voltage (V_in_) across the load resistor (R_P_) in a virtual ground configuration. It is important to note that the input voltage (V_in_) can also be applied from a third DC battery with a voltage regulator circuit. The analog signal across the input (V_in_ and ground) and output (pin 6 and ground) terminals were recorded by the DAQ system using a custom-written LabVIEW code. All ground cables were connected to the Aluminum box to keep the electrical noise low. The schematic of the above-mentioned electrical circuit is shown in Fig 1A. Two BNCs for connecting the load resistor (R_P_), a DB9 connector for sending and receiving the signal from DAQ, an On/Off DPST toggle switch connected to the batteries to power the amplifier, the circuit board, and a power LED are shown in the interior of the lab amplifier in Fig 1B. Simulations for these amplifiers were done using Tina-TI simulation tool. During cell experiments, the load resistor R_P_ is replaced by the micropore device. In Fig 1C, we show two lab amplifiers (L10 and L1 series) enclosed in an aluminum box, with the feedback resistor, bandwidth, and maximum measurable current labeled. A microfluidic device and a one rupee coin are shown in the image for size references. The description and cost of all the electronics parts used in making lab amplifiers are mentioned in S1 Table in S1 File. Borosilicate glass capillaries (Part # B100-50-10, Sutter Instruments) were pulled using a micropipette puller (P-2000, Shutter Instrument) and then polished using a flame polisher (MF-900, Micro Forge, Narishige) to fabricate the desired micropores (electro-fluidic device). The detailed information on the fabrication of the micropores (electro-fluidic device), sample preparation, and electro-fluidic measurements are presented in our previous work [48]. The schematic and brief details on how the micropore devices are prepared is included in the supplementary information file (see S2 Fig in S1 File). The two commercial amplifiers used in this work as a standard comparison are Dagan Chem Clamp and AM Systems Model 2400 (See S2 Table in S1 File for detail). Latex beads of different diameters (see text) were used as model cells for testing the signal-to-noise, throughput, and resolution. Events in the conductance traces are detected using atleast 1.5σ (1.5 times the standard deviation of baseline noise) thresholding from the baseline. DH5α strain of *E*. *coli* bacteria were grown overnight in Luria broth at 37°C, and 180 rpm overnight. Cells were washed with 1X-PBS (137 mM NaCl, 4.3 mM Na_2_HPO_4,_ 2.7 mM KCl_,_ and 1.47 mM KH_2_PO_4_ at pH 7.4) buffer before its cell volume measurements. Bacterial cells were stained with FM4-64 (Cat # T13320 Invitrogen) dye to a final concentration of 10 μM and imaged using Andor iXon DU-885K-CS0 camera (Oxford Instruments) and fluorescent lamp illumination on an IX-73 Olympus microscope with 100X objective. For testing the effect of alcohol on the cell volumes, 1000 μl of *E*. *coli* bacterial culture was incubated with 50 μl of ethanol (EtOH) (5% v/v) for 30 minutes before measurement.

## Results

### Amplifier characteristics

#### Current range

To measure the current range of our L1 series (gain = 0.001 mV/pA, R_F_ = 1 MΩ, C_F_ = 4.7 pF) and L10 series (gain = 0.01 mV/pA, R_F_ = 10 MΩ, C_F_ = 1.5 pF) amplifiers, we measure their I-V characteristics for different R_P_ load resistors. The I-V characteristics of L1A, L1B and D001-1 amplifiers (all with R_F_ = 1 MΩ) for R_P_ values of 50 kΩ, 100 kΩ, 500 kΩ, and 1 MΩ electrical resistors are shown in the Fig 1D.

The inset shows the I-V characteristics of L10A and L10B compared with commercial amplifiers D01-10 and AM10 (R_F_ = 10 MΩ) for R_P_ values of 0.5, 1, 5, and 10 MΩ electrical resistors. The current across R_P_ = 50 kΩ resistor with L1A, L1B, and D001-1 amplifiers saturates at ±10 μA, and for R_P_ = 500 kΩ the L10A, L10B, D01-10, and AM10 amplifiers saturate at ± 1000 nA. The current range is limited by the saturation voltage of the Op-amps in the amplifiers. The values of the current ranges for all the amplifiers are listed in S2 Table in S1 File.

*Absolute Error and Root Mean Square (RMS) Noise in Current*: Current was measured across load resistors of values 100 kΩ, 500 kΩ, 1 MΩ, 5 MΩ, 10 MΩ, 50 MΩ and 500 MΩ at voltages ± 200 mV, ± 400 mV, ± 600 mV, ± 800 mV, and ± 1000 mV. The absolute error percentages, as defined by Eq 1, in the current was calculated at all the measured voltages (Note: the current values in the saturation region (beyond measurable current range) were neglected for all the amplifiers):

$$Absolute\;Error\%=Abs\left(\frac{I_{Th}-I_{Meas}}{I_{Th}}\right)*100$$ (1)

**Fig 2:**
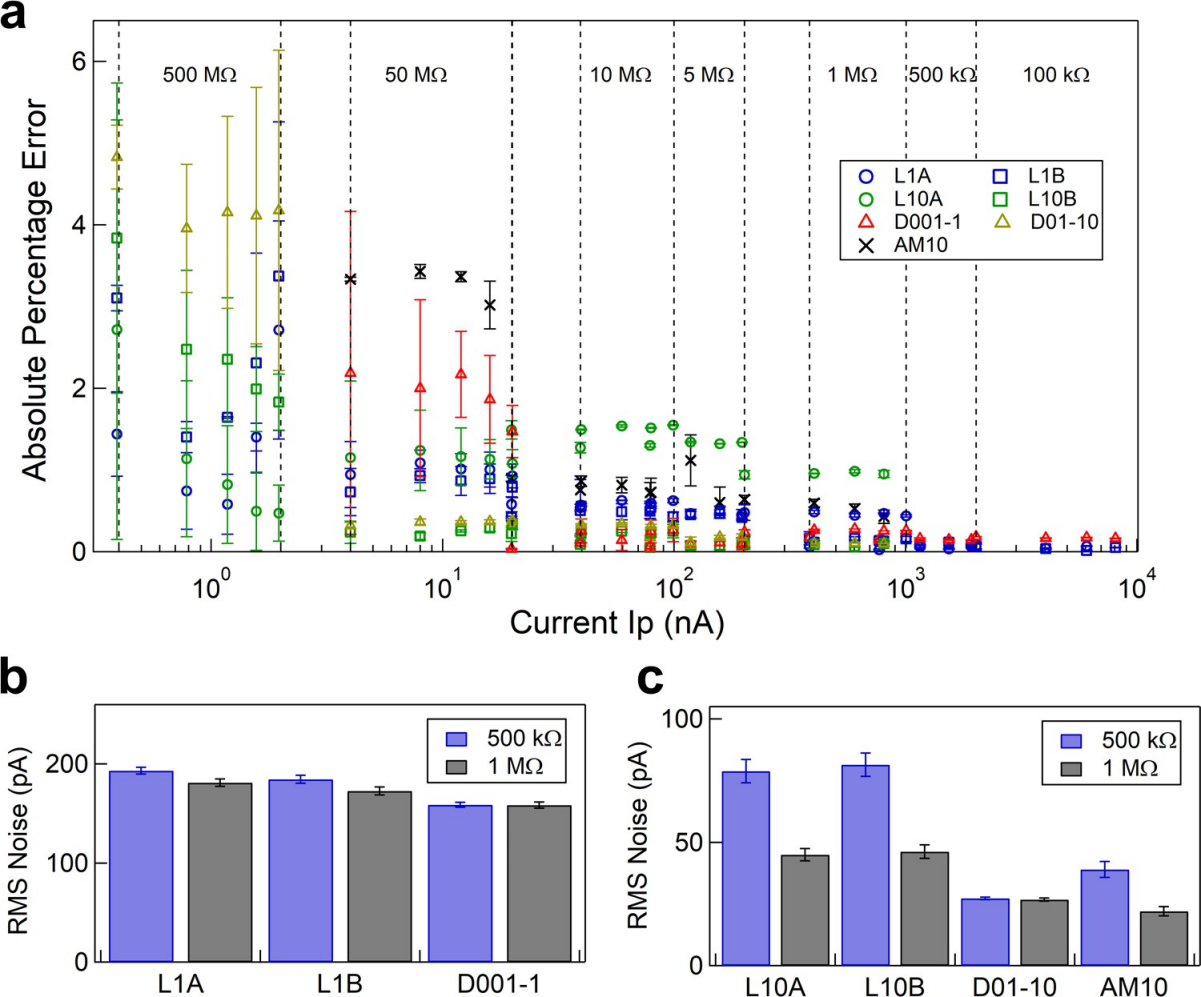
Comparison of absolute percentage error and RMS noise in current with different amplifiers. **a** Absolute error percentage in the measured current as defined in Eq (1), is plotted against the expected current for different amplifiers. Note that the current was measured at both positive and negative voltages to get an absolute mean current value and errorbars. Blue and green markers are for lab-amplifiers and red, yellow and black are for commercial amplifiers. **b** and **c** show RMS noise and the error bar values (at 1 kHz) in the current, measured at ± 300 mV for R_P_ = 500 kΩ (blue) and 1 MΩ (grey), by different amplifiers with feedback resistor (R_F_) values 1 MΩ (b) and 10 MΩ (c). A 600 ms current series was recorded at 100k sample/sec, 10 such sets at ± 300 mV were used for estimation of RMS noise and respective errorbars. Online version in colour.

Here, I_Th_ is the theoretically expected current, and I_Meas_ is the experimentally measured current for a known load resistor R_P_. Fig 2A shows a semi-log plot of the absolute percentage error vs. the theoretically expected current values for different lab and commercial amplifiers. The dotted regions in the plot show the electrical resistor used as the load. We note that the current measured by our lab amplifiers has an error of less than 6% and does as good as (or better than) the commercial amplifiers. The load resistance of our microfluidic devices used for cell volume measurements is in the range of ~1 MΩ, where we find the absolute percentage errors to be less than 1%. We next recorded the current at ± 300 mV for electrical resistors 500 kΩ and 1 MΩ (our experimental range) and measured the RMS noise in current at 1 kHz filter frequency. As seen in Fig 2B and 2C, in the experimental range of load resistances (R_P_), our lab amplifiers show RMS noise of < 200 pA (L1 series) and < 80 pA (L10 series).

The RMS noise values at other filter frequencies are shown in S3 and S4 Tables in S1 File. We note that the RMS noise values of our lab-amplifiers (comparable to the commercial amplifiers) and the large signal-to-noise (see later in Fig 4A) in the cell measurements demonstrates suitability of our devices for such measurements.

#### Bandwidth of lab amplifier

**Fig 3:**
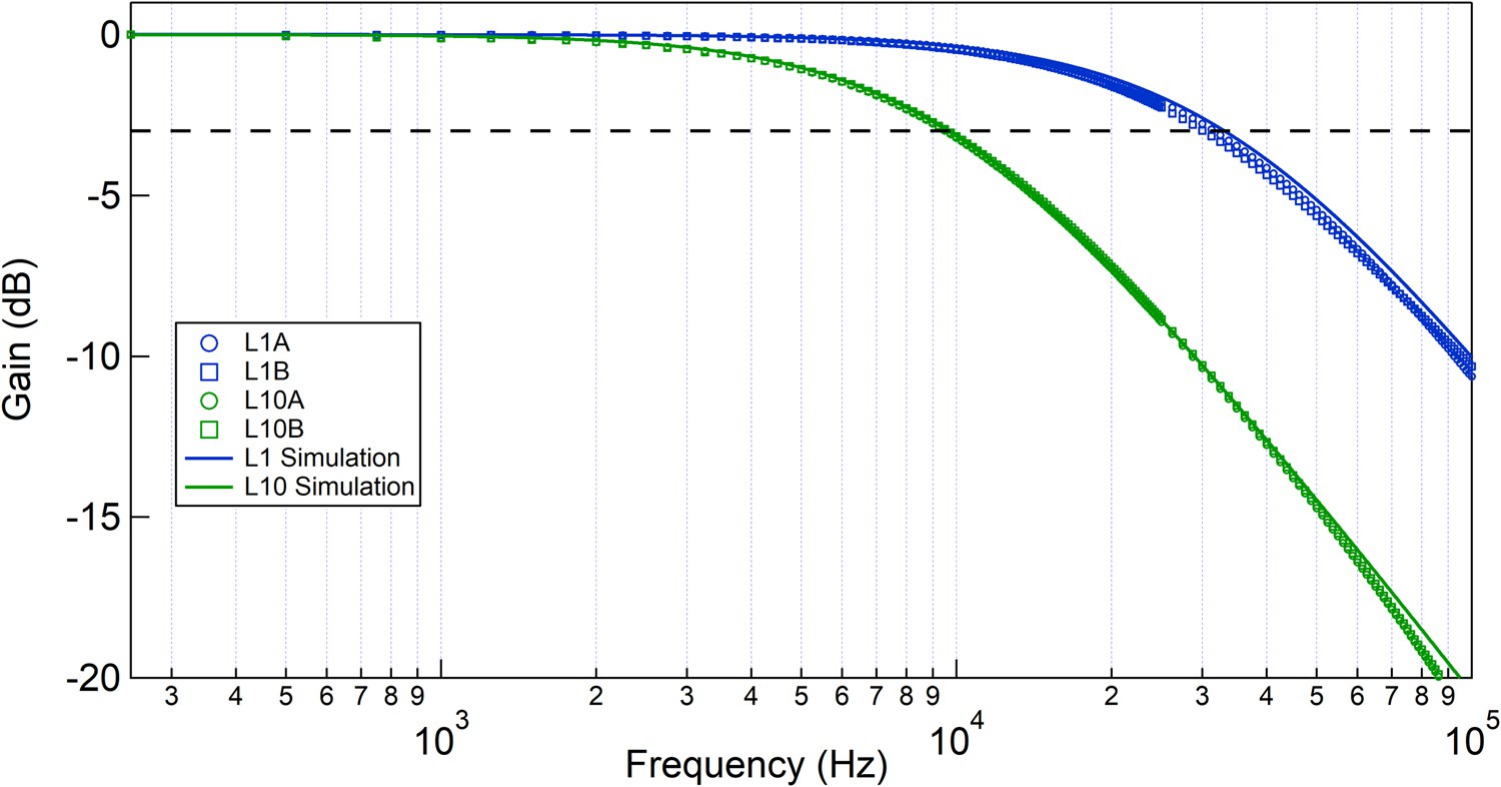
Bandwidth measurement of lab-amplifiers. Frequency response of the constructed amplifiers are shown here. The horizontal black dotted line is the 3dB drop in signal. A 500 kΩ resistor was used for all the measurements. L1 (A&B) and L10 (A&B) amplifiers show bandwidths of 30.9 ± 0.8 kHz, and 9.55 ± 0.05 kHz respectively. Online version in colour.

We next calibrated the frequency response of our lab amplifiers. The low-pass cutoff frequency decides the time response of the amplifier, which in turn decides the maximum throughput of cells that can be measured per second. The Gain (dB)-Frequency (Hz) response curves of different lab amplifiers (with R_P_ = 500 kΩ) is shown in Fig 3, where the black horizontal dotted line shows the -3dB decrease in the gain value. For this measurement, a data acquisition card (DAQ) with a maximum sampling rate of 2 MHz was used to apply a clean sine signal of 150 mV amplitude (V_in_) of different frequencies and then recorded the output sine signal (V_out_). The Gain (dB) was measured as $20\times \log\left(\frac{V_{out}}{V_{in}}\right)$, for each frequency.

The blue (circle- L1A, square- L1B) and green (circle- L10A, square- L10B) markers are experimental data, whereas the solid blue (L1) and green (L10) lines are simulation results of the lab amplifier frequency response. The y-axis is scaled so that maximum gain appears at 0 dB. The cutoff frequencies were estimated from the graph at the intersection point of the -3dB line (horizontal black dotted line) and the response curve, and the values are listed in S2 Table in S1 File. We show that L1-series amplifiers have a relatively higher bandwidth of ~30 kHz, whereas the L10 series amplifiers are of ~10 kHz bandwidth.

### Translocation measurement

#### Detection of model cells using lab-amplifiers and electro-fluidic devices

**Fig 4:**
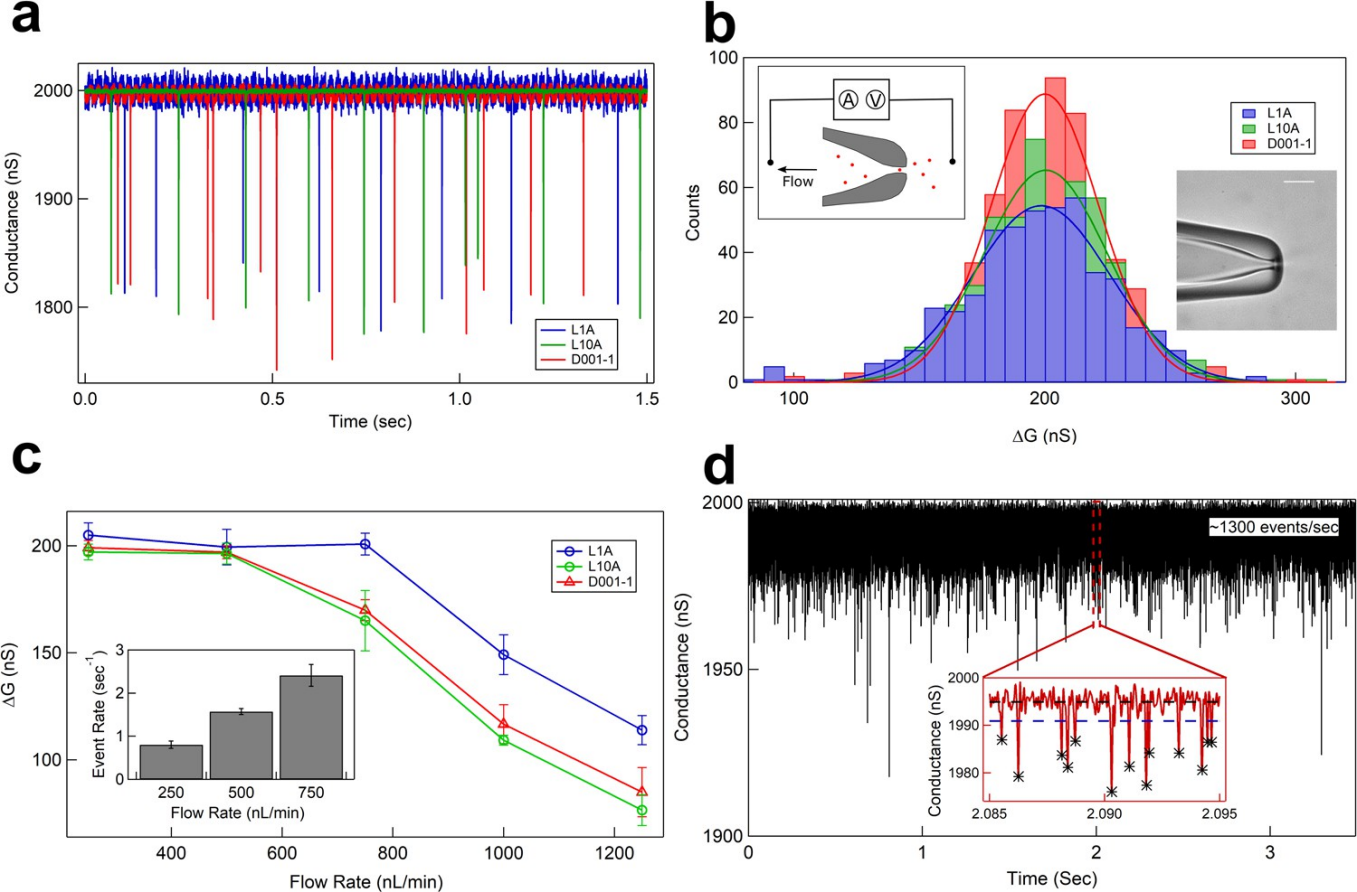
High throughput detection of model cells using lab amplifiers. **a** The plot shows 1.5 sec time series of open pore conductance baseline and the translocation events of 4.98 μm beads translocating through a 6.8 μm micropore device as measured using the L1A (blue), L10A (green) and D001-1 (red) amplifiers. Measurement was done at constant fluid flow of 500 nL/min. **b** ΔG histograms of detection events measured from the three different amplifiers are shown. Inset-left shows the schematic of the micropore experiment where the fluid flow is generated by a syringe pump and pore current across the micropore is measured by the amplifier. Inset-right shows optical microscope image of a typical micropore fabricated from a glass capillary. **c** Shows the mean signal (ΔG) of the translocation events as a function of fluid flow rates measured using different amplifiers. The inset shows the change in event rates for different flow rates for L1A amplifier. **d** Demonstrates typical detection throughput of our lab amplifier (L10A). 4.06 μm beads were detected through the same 6.8 μm micropore device at a fluid flow of 50 μL/min with the average event rate of 1308.4 particles/sec. The inset shows a 10 ms zoom of the times series and the detected events are marked by black stars. The baseline and the detection thresholds are shown by black and blue dashed lines, respectively. Online version in colour.

We next show cell volume detection capabilities using model cells measured with our electro-fluidic device and lab-amplifiers. Our devices make electrical measurements of cell volumes, with single-cell resolution, as individual cells translocate through the micropore under an applied flow and electrical potential [48]. Fig 4A shows 1.5 sec long time series of translocation events caused by 4.98 μm beads translocating through a 6.8 μm micropore device as measured using the L1A (blue), L10A (green) and D001-1 (red) amplifiers. Schematic of a typical micropore (inset (right)) and a measurement device (inset (left)) is shown in Fig 4B. Translocation of the model cells was maintained by a 500 nL/min constant fluid flow and an applied potential of 300 mV. Ions in the 1X-PBS buffer move across the unobstructed micropore resulting in an open pore conductance. As the model cells translocate through the micropore, they block the pore conductance (ΔG (nS)) for the duration of translocation (dwell time, Δt (ms)). An electrical conductance drop (ΔG) signals the translocation of a single cell and is directly proportional to the cell volume [48, 51, 52, 54]. In each dataset, we collect electrical conductance blockage events for 500 cells or more that translocate through the pore to measure population average cell volumes and changes in it, if any. Fig 4B shows the population average of ΔG histograms. We show high signal-to-noise measurements on the same device and sample using the L1 and L10 lab amplifiers and compare it with the D001-1 commercial amplifier. The identical and overlapping histograms in Fig 4B show numerically identical ΔG values for the model cell population when measured using any amplifier.

**Fig 5:**
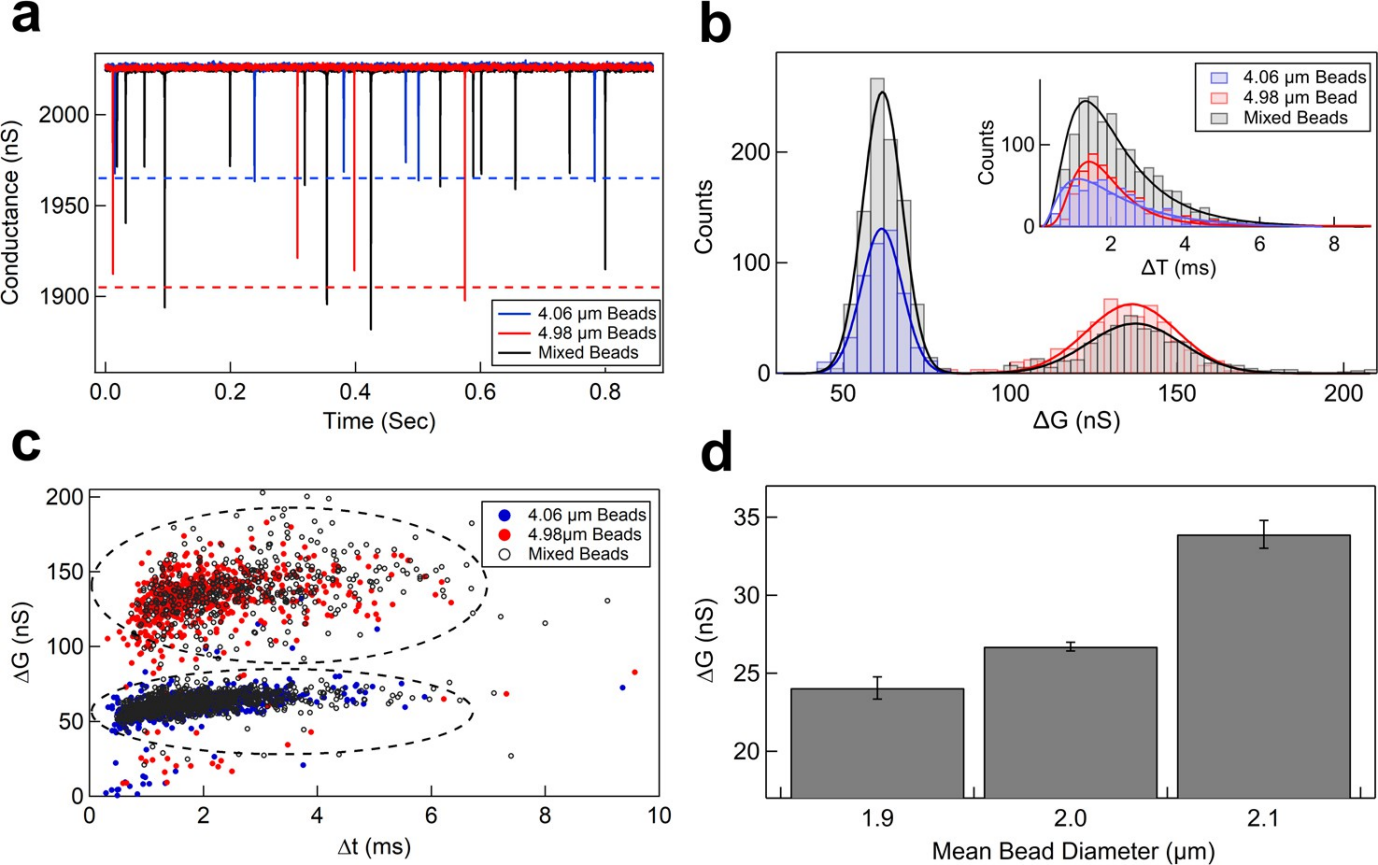
Resolving and quantifying mixed sample population. **a** shows translocation events for 4.98 and 4.06 μm beads translocating through a 7.6 μm micropore device measured using L10A amplifier. Blue and red data is for individual sample measurements and black data is for a mixed beads sample. **b** Shows the Δt (inset) and ΔG histograms of individual and mixed sample translocation experiments with their respective lognormal and Gaussian fits. **c** The ΔG-Δt scatter plot shows the two populations of the beads. The result of the quantification of the population ratios of mixed beads is shown in Table 1. Online version in color. **d** Bar plot of ΔG values (N ≥ 3 data sets for each bead size) for beads with diameters 1.9, 2.0, and 2.1 μm translocating through a 3.0 μm micropore device.

We demonstrate the effect of the bandwidth of the amplifier on the measured signal by translocating the 4.98 μm latex beads through the same 6.8 μm micropore at different fluid flow rates. At larger flow rates, the event rate of cells translocating through the device increases dramatically (see Fig 4C inset). The ΔG values of model cells measured at different flow rates are shown in Fig 4C. We show that the amplifiers maintain the correct ΔG values up to certain throughput, after which, although the cells are detected successfully, the measured ΔG values drops. This is due to the low pass filtering of the amplifiers. We show in Fig 4C that the L1 amplifier (30 kHz bandwidth) maintains the measured ΔG values up to much higher flow rates (750 nL/min of fluid flow), whereas for other amplifiers (L10 and D00-1, both with 10 kHz bandwidth) ΔG values drop after 500 nL/min of fluid flow. The choice of amplifier bandwidth is important to accurately quantify the translocation dynamics. For the translocation of particles and cells used in our study, 10 kHz bandwidth was found sufficiently high so as not to affect the ΔG conductance drop values (see S3 Fig in S1 File). Hence, the amplifier and the measurements were optimized to be recorded by 10 kHz bandwidth so that the signal doesn’t experience any distortion at the fluid flow rates used for experiments in this paper. Our amplifiers can be used in two different modes. The sensitivity mode (see Fig 4B and later in Fig 5) where the translocation speeds are more controlled and the current drop amplitudes correspond to cell volumes and the high-throughput mode where the cells are detected at a very high speed. We demonstrate the high-throughput detection of model cells in Fig 4D. We use the 6.8 μm micropore device to translocate 4.06 μm model cells at a fluid flow of 50 μL/min and demonstrate a detection event rate of ~1300 particles per second. In Fig 4D, we show a conductance trace of ~3.5 seconds (using an L10A amplifier), showing such a high detection throughput. The inset of Fig 4D shows a 10 ms zoom of the time series along with the baseline (dotted black line), detection threshold (dotted blue line), and the detected events are marked (black stars).

#### Resolving and quantifying mixed sample population

We next demonstrate the resolution of our measurement electronics by resolving model cells that differ in diameter by 1 μm. In Fig 5A, we show the conductance blockade events corresponding to model cells of diameters 4.06 μm (blue), 4.98 μm (red), and a mixture of both 4.06 and 4.98 μm (black) translocating through a 7.6 μm micropore device. A comparison of the translocation time (Δt) and change in conductance (ΔG) along with the respective lognormal (inset) and Gaussian fits for the same samples are shown in Fig 5B respectively. Although the two different model cells are not resolvable in the Δt histogram, they are very well resolved in the ΔG histograms (red and blue histograms). In the mixed sample experiment, the ΔG histogram shows two clear well-separated populations (Fig 5B, black histogram) corresponding to individual populations of 4.06 μm and 4.98 μm model cells. In Fig 5C, we also show the ΔG-Δt scatter plot that also clearly shows the presence of two distinct cell size populations.

We further quantify the mixed sample data by comparing the number-ratio of events in each population histogram to the number-ratio in which the mixed sample was prepared. Since there is a random chance of any type of cell to translocate through the pore, the two ratios must be equal. The two populations were isolated by fitting Gaussian peaks (with no y-offset) to the histograms (Fig 5B) and estimating number of events in each peak. The quantitative data for the mixed sample is shown in Table 1 (average of three independent experiments). The first column of each set is the ΔG value and the spread in the histogram, and the second column is the number of events corresponding to the respective latex beads. The last column shows the sample concentration used in the experiment from the bead manufacturer’s numbers. The 4.06 and 4.98 μm beads were mixed in a known ratio of 2.5:1, the last row of the Table 1 shows the event ratio measured from the translocation experiment. The average of the event ratio from three sets gives us (2.49 ± 0.08): 1, which is remarkably close to the ratio in which the beads were mixed. Thus, our devices, along with our custom amplifiers, may be used in the field for testing multiple types of cells that differ in size and concentrations. In Fig 5D, we demonstrate the resolution capabilities of our system by detecting changes in particle volumes as low as 0.6 femtoliter. The bar plot in this figure shows comparison of measured ΔG values of 24.1 ± 0.7, 26.7 ± 0.3 and 33.9 ± 0.9 nS respectively, for beads of diameter 1.90 ± 0.04, 2.00 ± 0.04, and 2.1 ± 0.3 μm translocating through a 3.0 μm micropore device. In our previous work, we have shown similar contrast between particles using commercial amplifiers [48], Fig 5D demonstrates that in terms of resolutions, our custom made lab-amplifier compares very well to the commercial amplifiers.

**Table 1:** Quantification of mixed samples.

Bead Size (μm)	Set1		Set2		Set3		Manufacturer’s Data (# x 10^9^/μl)
	ΔG (nS)	# Events	ΔG (nS)	# Events	ΔG (nS)	# Events	
**4.06**	60 ± 8	940	60 ± 8	964	60 ± 9	1468	**1.365**
**4.98**	140 ± 20	397	140 ± 20	375	140 ± 20	583	**0.547**
**Events Ratio**	2.37: 1		2.57: 1		2.52: 1		**2.5: 1**

ΔG and number of events detected corresponding to 4.06 and 4.98 μm latex beads in a 7.6 μm micropore device for three sets of translocation experiments is shown in the table. The 4.06 and 4.98 μm beads mixed in a known ratio of 2.5:1, and the experimental values of the detected ratio for 3 sets are mentioned in the last row.

#### Effect of alcohol on bacterial cell volume

**Fig 6:**
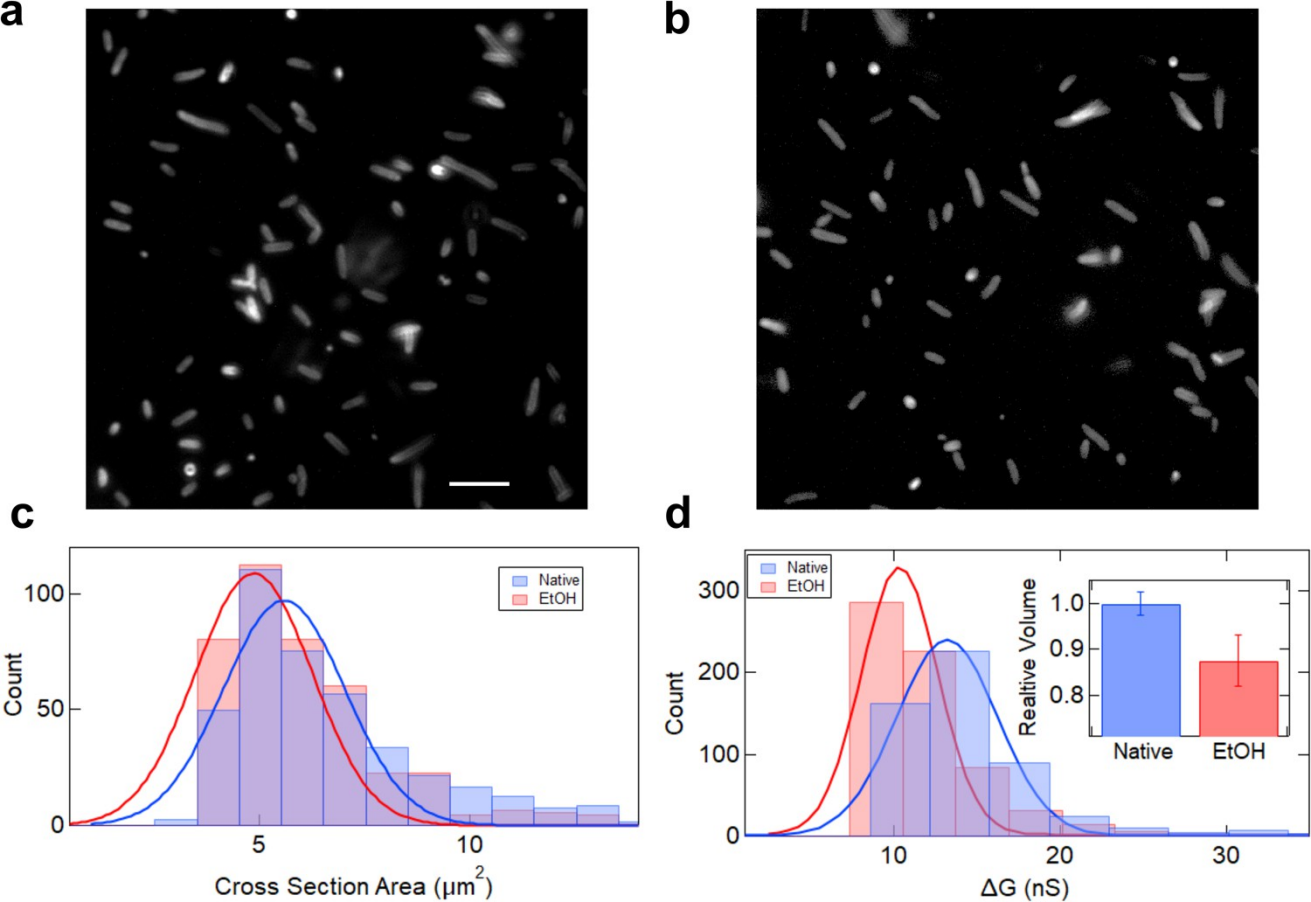
Measurement of changes in bacterial relative volumes using lab amplifier. **a** and **b** are flourescent images of DH5α strain of E. coli bacteria in 1X PBS buffer with and without ethanol exposure respectively (scale bar is 10 μm). The histogram in **c** shows the projected area estimated from the fluorescent images for bacterial cells. **d** compares the ΔG histograms of E. coli cells measured in a 2.1 μm micropore device. The inset is a bar plot showing relative change in cell volumes when cells are treated with 5% (v/v) ethanol when compared to their native state in 1X PBS buffer (average of 4 experiments). Online version in colour.

Finally, we apply our measurement system with the lab amplifier (L10 series) to quantitatively understand the effect of ethanol on bacterial cell physiology. The FM4-64 stained fluorescent images of the cells suspended in 1X PBS buffer, exposed with and without ethanol, are shown in Fig 6A and 6B, respectively. Particle analyzer plugin of ImageJ software was used post thresholding to estimate the projected area for the bacteria in the fluorescent images. The histogram in Fig 6C shows the projected area estimated from ~ 400 bacteria cells each with and without alcohol exposure. The Gaussian fit to the histograms estimates the values as 5.09 ± 0.14 μm^2^ and 4.87 ± 0.12 μm^2^ for native and ethanol exposed state of bacteria, respectively. The changes in the cell’s projected area are hard to quantify when measured using imaging techniques. The electrical measurement data, on the other hand, shows excellent contrast in the cell volumes of these two cell populations. The ΔG histogram in Fig 6D shows the change in signal for bacterial translocation through a 2.1 (see S1 Fig in S1 File for image) μm micropore device. The ΔG value from the Gaussian fits are 11.4 ± 4.3 (Blue- Native) and 8.7 ± 3.2 nS (Red- 5% EtOH exposed). Relative change in the ΔG values directly corresponds to the relative change in cell volume [48] using the following equation:

$$Relative\;volume\;(RV)=\frac{\Delta G_{Sample}}{\Delta G_{Control}}$$ (2)

Here, the ΔG_Control_ is the measured value for the native cells, and ΔG_Sample_ is the measured value for cells exposed to 5% ethanol. The bar plot in the inset shows the relative change in volume when the cells are treated with ethanol for 30 minutes, with respect to their native state. We note a 12.6% reduction (average of 4 experiments) in bacterial cell volume upon exposure to just 5% ethanol solution. It is important to note that all the pulse measurements are made relative to the baseline and the reduction in bacterial size is solely due to the shrinkage of cells in the presence of ethanol and not due to the decrease in absolute conductivity of the solution as shown in S4A Fig in S1 File. In order to demonstrate this, we translocate 4.0 μm beads through a 6.1 μm diameter micropore (See S4B Fig in S1 File) and, 1.9 and 2.1 μm beads through a micropore of 3.0 μm diameter (See S4C and S4D Fig in S1 File) in suspension buffers containing different concentrations of ethanol. The constant ΔG values for different ethanol concentrations in S4B–S4D Fig in S1 File for solid latex particles confirms that the change in conductance measured for bacterial cells is solely due to shrinkage of cells. We have demonstrated the ability of our electro-fluidic device, along with the custom-made lab-amplifiers, to detect cells with high throughput and measured changes in cell volumes with high resolution.

## Conclusion

In this work, we present an in-field point-of-care compatible system which is cost-effective, portable, and has simple-to-build electronics. This system consists of electrical measurements using a microfluidic device and a lab-made amplifier (L1 and L10 series). The lab amplifier is characterized for the maximum current range, absolute error, RMS noise in the measured current signals, and bandwidth. All the electrical characterization demonstrate that the lab amplifiers perform comparable to the commercially available amplifiers. Using our lab amplifiers, we showed high throughput detection of up to 1300 cells/sec, demonstrating its potential as a cell counter device. We demonstrate the high resolution of our system by measuring cell size differences down to 100 nm. Estimation of absolute volumes of the translocating particles from the measured ΔG values, require detailed modeling of particle and pore’s internal geometry. However, the relative change in cell volume, upon exposure to ethanol, is straight forwardly estimated provided the particle and pore shape remains the same. We finally presented a real-world measurement example by quantitatively measuring small physiological changes in the bacterial cells upon mild alcohol exposure. We report a 12.6% decrease in bacterial volume upon ethanol exposure of 5% v/v. The quantitative knowledge of cell shrinkage upon mild alcohol exposure is a vital step in understanding the adaptive behaviour of bacterial cells which they use to maintain their cellular integrity against certain alcohol-based disinfectants. The measurement of changes in bacterial volume is presented here as proof of concept for sensitive detection of volume-change in cells, using our lab-amplifiers. The simple design of our lab amplifier, its low cost, portability, high throughput, and resolution makes it a promising device for large-scale population screening applications in hospital and in-field rural areas. We envisage possible application of this system for red blood cell-based population screening for disease that directly affect cell size, such as, malaria and sickle cell anemia.

## Supporting information

S1 File Supporting information file contains supplementary tables and figures as mentioned in the main text. (DOCX)

S1 Fig (TIF)
