# Supplementary material for: Customized low-cost high-throughput amplifier for electro-fluidic detection of cell volume changes in point-of-care applications
Source: PLoS One. 2022 Apr 20;17(4):e0267207. doi: 10.1371/journal.pone.0267207 (PMC9020695; doi:10.1371/journal.pone.0267207)
Supplement: S1 File — (DOCX) [file pone.0267207.s001.docx]

Customized low-cost high-throughput amplifier for electro-fluidic detection of cell volume changes in point-of-care applications

Saurabh Kaushik^#^, Prabhakaran Selvanathan^#^ & Gautam Vivek Soni*

Raman Research Institute, Bangalore – 560080 INDIA

SUPPLEMENTARY INFORMATION FILE

This PDF file includes:

Tables S1-S4

Figures S1-S4

| S.no | **Item Description** | **Quantity** | **Price/pc (INR)** | **Price (INR)** |
| --- | --- | --- | --- | --- |
| 1 | IC AD820 | 1 | 475 | 475 |
| 2 | Electrical Resistors | 1 | 11.12(1 MΩ)  2.80 (10 MΩ) | 11.12(1 MΩ)  2.80 (10 MΩ) |
| 3 | Ceramic Capacitors | 1 | 64 | 64 |
| 4 | DB9 Connector Cable | 1 | 362 | 362 |
| 5 | DB9 Adaptor | 1 | 144 | 144 |
| 6 | LED | 1 | 9 | 9 |
| 7 | Aluminium Enclosure | 1 | 899 | 899 |
| 8 | Female BNCs | 2 | 94 | 188 |
| 9 | Crocodile Clips | 2 | 55 | 110 |
| 10 | Breadboard | 1 | 275 | 275 |
| 11 | DC Batteries | 2 | 259 | 518 |
| 12 | Connecting wires | 5 meters | 35 | 35 |
| 13 | ON/OFF DPST toggle switch | 1 | 304 | 304 |
| **TOTAL** | | | | **3394** |

**Table S1: Total Expenditure.** The table shows the items used to make the amplifier, the quantity required and the respective price to buy the items. Our total cost of INR 3394 (about USD 50) is about 100 times lower than the commercial amplifiers.

| **Amplifier Type**  **(Commercial or Lab)** | **Legend** | **R_F_ (MΩ)** | **Range (nA)** | **Measured RMS Noise @ 1 kHz (pA)** | **Bandwidth**  **(kHz)** |
| --- | --- | --- | --- | --- | --- |
| Lab Amp | L1A | 1 | ±10000 | 193 ± 3 | 31.6 |
| Lab Amp | L1B | 1 | ±10000 | 185 ± 4 | 30.1 |
| Commercial Amp-1 | D001-1 | 1 | ±10000 | 159 ± 3 | 10 |
| Lab Amp | L10A | 10 | ±1000 | 79 ± 5 | 9.5 |
| Lab Amp | L10B | 10 | ±1000 | 82 ± 5 | 9.6 |
| Commercial Amp-2 | D01-10 | 10 | ±1000 | 27.3 ± 0.5 | 10 |
| Commercial Amp-3 | AM10 | 10 | ±1000 | 39 ± 3 | 10 |

**Table S2: Summary of the comparison between all the amplifiers.** The column-1 is the type of amplifier used (commercial or lab) with the respective legend code in the column-2. The values in Column-3, 4, 5 and 6 are feedback resistor (R_F_), the range of measurable current range (at ± 10 volts V_CC_), RMS noise in the measured current for a 500 kΩ electrical resistor at 1 kHz software filter and the bandwidth of the amplifier respectively.

| **Filter Frequency** | **L1A** | **L1B** | **D001-1** | **L10A** | **L10B** | **D01-10** | **AM10** |
| --- | --- | --- | --- | --- | --- | --- | --- |
| **Unfiltered** | 1372 ± 7 | 1119 ± 8 | 1191 ± 6 | 177 ± 2 | 175 ± 2 | 121.2 ± 0.3 | 63 ± 2 |
| **50 kHz** | 1372 ± 7 | 1120 ± 8 | 1190± 6 | 177 ± 2 | 175 ± 2 | 121.3 ± 0.3 | 63 ± 2 |
| **30 kHz** | 1009 ± 5 | 858 ± 7 | 928 ± 5 | 155 ± 3 | 157 ± 2 | 96.4 ± 0.3 | 59 ± 2 |
| **20 kHz** | 837 ± 5 | 717 ± 6 | 779 ± 4 | 144 ± 3 | 146 ± 3 | 81.9 ± 0.2 | 55 ± 2 |
| **10 kHz** | 637 ± 5 | 531 ± 5 | 564 ± 4 | 126 ± 3 | 129 ± 3 | 61.8 ± 0.2 | 50 ± 2 |
| **5 kHz** | 461 ± 4 | 383 ± 4 | 385 ± 3 | 109 ± 4 | 112 ± 3 | 45.9 ± 0.3 | 47 ± 3 |
| **1 kHz** | 193 ± 3 | 185 ± 4 | 159 ± 3 | 79 ± 5 | 82 ± 5 | 27.4 ± 0.5 | 39 ± 3 |

**Table S3:** The table shows the RMS noise values (pA) in current measurements for L1, L10 series lab amplifiers and D001-1, D01-10 and AM10 commercial amplifier at 1, 5, 10, 20, 30, 50 kHz filtered and unfiltered frequency. The current measurements were made at ± 300 mV with a 500 kΩ load resistor (R_P_).

| **Filter Frequency** | **L1A** | **L1B** | **D001-1** | **L10A** | **L10B** | **D01-10** | **AM10** |
| --- | --- | --- | --- | --- | --- | --- | --- |
| **unfiltered** | 1320 ± 10 | 1102 ± 9 | 1180 ± 7 | 141 ± 1 | 126 ± 2 | 122.2 ± 0.5 | 50.2 ± 0.8 |
| **50 kHz** | 1318 ± 9 | 1102 ± 9 | 1181 ± 7 | 141 ± 1 | 126 ± 2 | 122.2 ± 0.5 | 50.2 ± 0.8 |
| **30 kHz** | 969 ± 8 | 842 ± 7 | 919 ± 6 | 116 ± 1 | 106 ± 2 | 96.6 ± 0.4 | 44.7 ± 0.9 |
| **20 kHz** | 803 ± 7 | 702 ± 6 | 771 ± 5 | 102 ± 1 | 95 ± 2 | 82.1 ± 0.4 | 39 ± 1 |
| **10 kHz** | 608 ± 6 | 516 ± 5 | 559 ± 4 | 84 ± 1 | 80 ± 2 | 61.7 ± 0.3 | 32 ± 1 |
| **5 kHz** | 436 ± 4 | 370 ± 4 | 381 ± 3 | 68 ± 2 | 67 ± 2 | 45.5 ± 0.4 | 29 ± 1 |
| **1 kHz** | 181 ± 4 | 173 ± 4 | 159 ± 3 | 45 ± 2 | 46 ± 3 | 26.8 ± 0.6 | 22 ± 2 |

**Table S4:** The table shows the RMS noise values (pA) in current measurements for L1, L10 series lab amplifiers and D001-1, D01-10 and AM10 commercial amplifier at 1, 5, 10, 20, 30, 50 kHz filtered and unfiltered frequency. The current measurements were made at ± 300 mV with a 1 MΩ load resistor (R_P_).


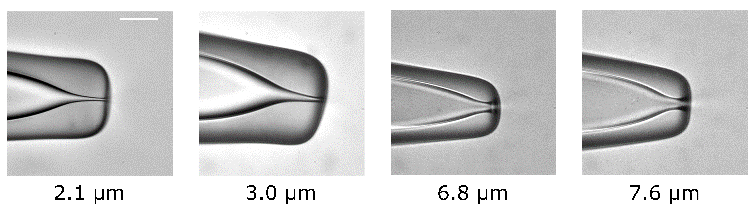


**Figure S1:** Optical Image library of micropores used in this paper. Scale bar (30 μm) is common to all images.


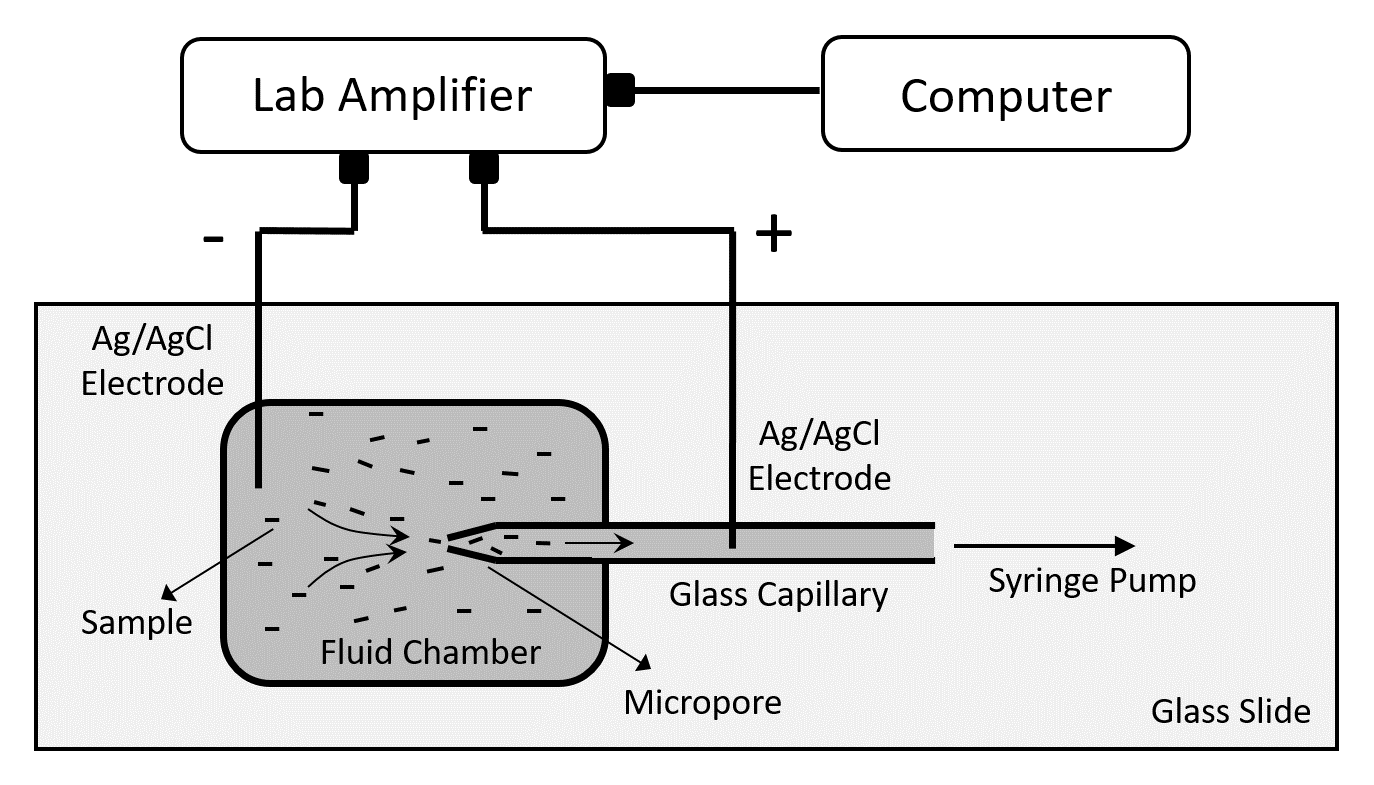

**Figure. S2: Schematic of the micropore setup.** A glass micropore is mounted in the fluid chamber containing the suspension buffer and sample. The syringe pump is connected to the glass capillary to create a stable fluid flow to translocate the sample through the micropore. The translocation current signals are acquired using two Ag/AgCl electrodes, one immersed into the buffer in the fluid chamber and other inserted in the tubing. These electrodes are connected with the lab amplifier, which is controlled using a custom made LabVIEW code. The entire micropore device unit is mounted on a glass slide.


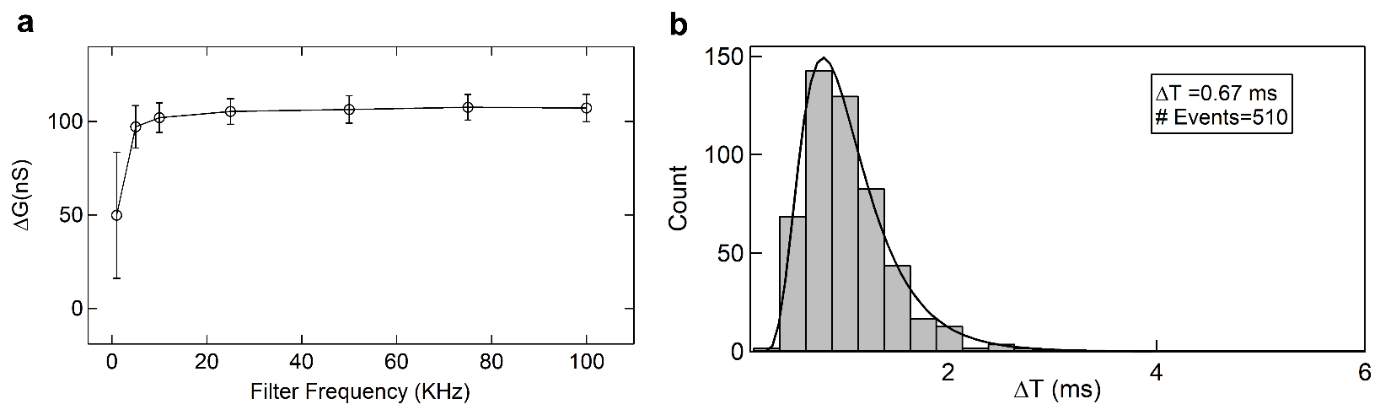


**Figure S3: a** Plot of ΔG values for 4.98 µm beads translocating through an 8.3 µm micropore data measured at 100 kHz (using commercial Axopatch 200B amplifier, flow velocity 500 nL/min) and digitally filtered at different filter frequencies. **b** ΔT histogram of 2.1 µm beads translocating through a 3.0 µm micropore data with mean translocation times of 0.67 milliseconds. Solid line is log-normal fit to the distribution.


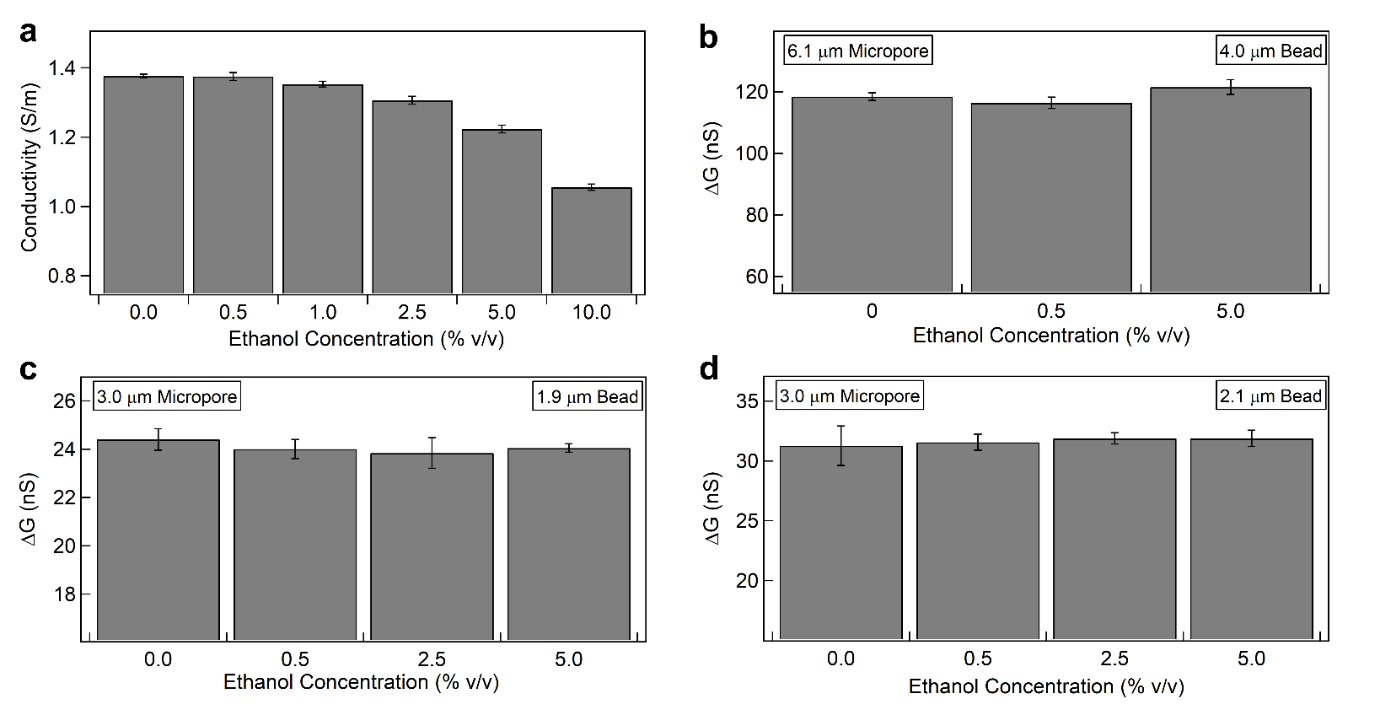


**Figure S4: Demonstration of constant ΔG with changing ethanol concentration.** **a** Absolute conductivity of phosphate saline buffer with different ethanol concentrations. **b** Bar plot of ΔG values for 4.0 µm beads translocating through a 6.1 µm micropore device with different ethanol concentration in the suspension buffer. **c** and **d** Bar plot of ΔG values for 1.9 and 2.1 µm beads translocating through a 3.0 µm micropore device with different ethanol concentration in the suspension buffer respectively.
