# Supplementary figures and images for: Customized low-cost high-throughput amplifier for electro-fluidic detection of cell volume changes in point-of-care applications
Source: PLoS One. 2022 Apr 20;17(4):e0267207. doi: 10.1371/journal.pone.0267207 (PMC9020695; doi:10.1371/journal.pone.0267207)

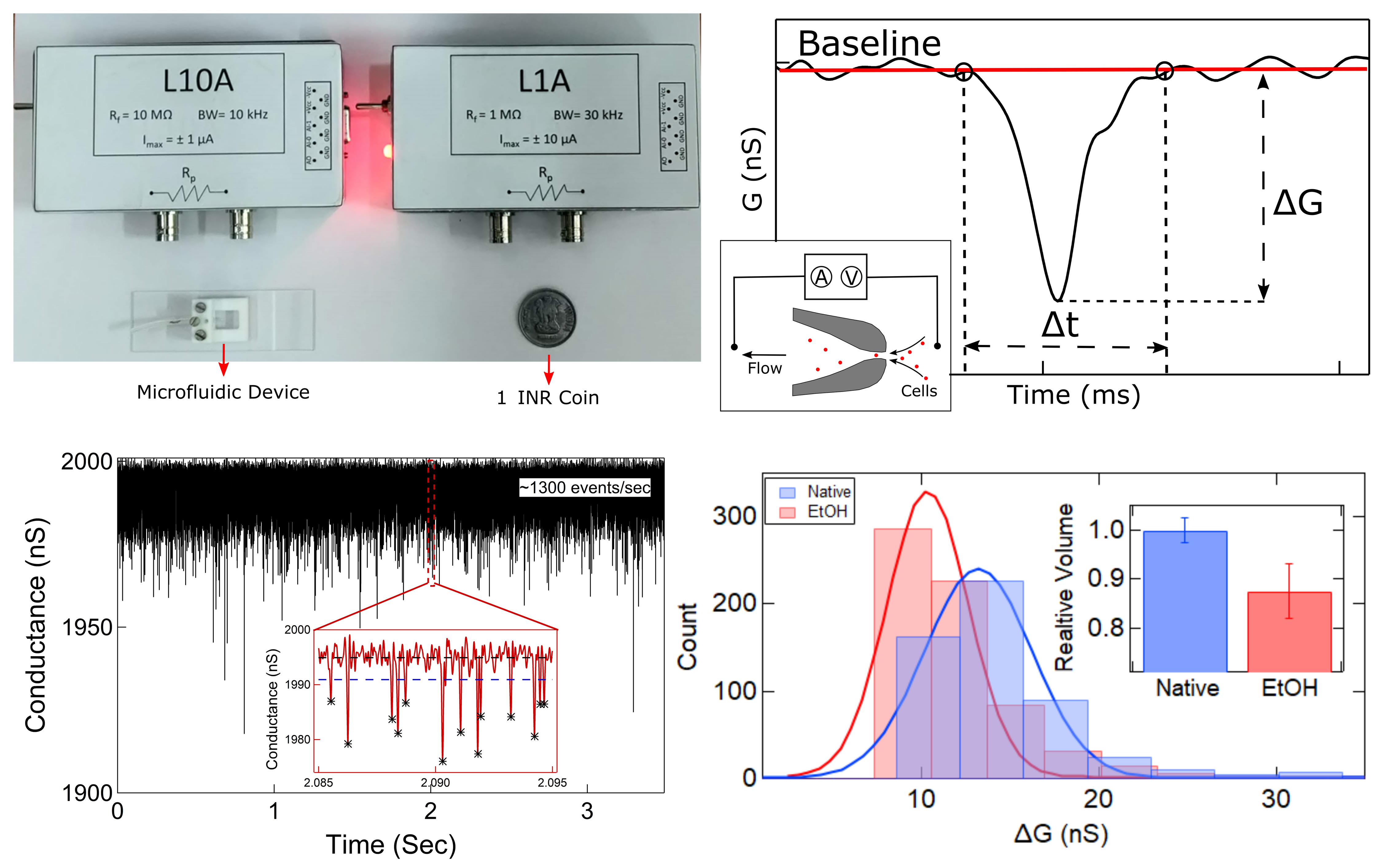

Supplement: S1 Fig — (TIF) [file pone.0267207.s002.tif]
